# Mineralogical and Geochemical Nature of Calcareous Vineyard Soils from Alcubillas (La Mancha, Central Spain)

**DOI:** 10.3390/ijerph17176229

**Published:** 2020-08-27

**Authors:** Raimundo Jiménez-Ballesta, Sandra Bravo, Jose A. Amorós, Caridad Pérez-de los Reyes, Rosario García-Giménez, Pablo Higueras, Francisco J. García-Navarro

**Affiliations:** 1 Department of Geology and Geochemistry, Autonoma University of Madrid, 28049 Madrid, Spain; rosario.garcia@uam.es; 2 Higher Technical School Agricultural Engineers of Ciudad Real, University of Castilla-La Mancha, 13071 Ciudad Real, Spain; sandra.bravo@uclm.es (S.B.); joseangel.amoros@uclm.es (J.A.A.); caridad.perez@uclm.es (C.P.-d.l.R.); fcojesus.garcia@uclm.es (F.J.G.-N.); 3 Institute Applied Geology, University of Castilla-La Mancha, 13400 Almadén, Spain; pablo.higueras@uclm.es

**Keywords:** clay mineralogy, geochemistry, trace elements, spatial distribution, La Mancha

## Abstract

The mineralogical and geochemical patterns of calcareous vineyard soils located in Alcubillas (La Mancha, Central Spain) have been evaluated; also their variability has been studied. The information provided by this study supports the assessment of geochemical spatial variability, the origin of these soils, their elements and the factors that control their distribution. The presence of quartz, calcite, feldspar and, in particular, illite and kaolinite is due to their inheritance from surrounding lithologies (and pedological processes), which mostly include limestones, marls and other sedimentary rocks, as well as metasedimentary rocks of Hercynian origin. Furthermore, since the presence and accumulation of certain trace elements in vineyard soils is a relevant global hazard (in particular with respect to wine production quality), the spatial distributions of Ba, Cr, Cu, Pb, Rb, Sr, V and Zr (carried out using geostatistical techniques and geometry-based interpolation methods) were investigated in order to determine the origin of these trace elements. The presence of these elements can be interpreted as being due to geogenic, pedogenic and, in certain cases, anthropic influences. The nature of certain agricultural practices, including the use of fertilizers, phytosanitary products and machinery, could explain the local increases in some trace element contents.

## 1. Introduction

It is widely accepted that, in a vineyard, the geology–bedrock–soil–vine system helps to explain the typicity of wine from a particular zone or terroir. However, few studies concerning the importance of local soil mineralogy and geochemistry have been published [1,2]. In fact, there is a lack of information about the mineralogy, concentrations and spatial distribution of trace elements in red calcareous vineyard soils, which are extensive in many subregions of the Mediterranean area. These soils typically include argillic horizons, and they often also include calcic or petrocalcic horizons [3,4]. The main soil-forming processes responsible for the genesis of these red Mediterranean soils include clay illuviation and rubefaction [5,6,7]. However, the latter authors did not identify clay coatings and, in addition, pedogenic carbonates occur frequently in these soil types, although their role is not fully understood.

Moreover, the presence of trace elements—particularly heavy metals or potentially toxic elements (PTEs)—in agricultural soils (including red soils) has been addressed in many regions of the world [8,9,10,11,12,13,14,15,16]. The presence of such elements represents a significant and general environmental problem, due to the risk of these elements entering the food chain. The presence of these elements is clearly also a risk to environmental security, due to the possibility of leaching and infiltration into groundwater [17]. These concerns are also applicable to vineyard soils.

The spatial variability maps of elements provide a very nice visual representation of their geographic distribution, and this helps us to understand the variability of such elements in relation to other characteristics of the studied area. The maps allow the recognition of source areas for the elements in question and ultimately provide a powerful tool to improve environmental assessment and management [18,19]. Geostatistical analysis, combined with GIS data, has frequently been used to answer these questions since it allows the values of an environmental variable to be estimated at unsampled locations. The application of geostatistics to data obtained from geochemical, pedological or anthropological processes can provide useful information about soil contamination, as shown by [8,20,21,22,23], amongst others.

Given the exceptional geomorphological, agro-ecological and climatic conditions of some regions in the Mediterranean environment, the research reported here was carried out on red calcareous soils of Alcubillas, a wine-producing area in La Mancha (Central Spain) that has hosted vineyards for many years. The main goals of this study were the characterization of the mineralogical and geochemical nature of these soils and to elucidate the spatial distribution of a number of trace elements (i.e., Ba, Cr, Cu, Pb, Rb, Sr, V and Zr). The final goal was to determine the possible sources of these elements.

## 2. Materials and Methods

### 2.1. Study Area

**Figure 1:**
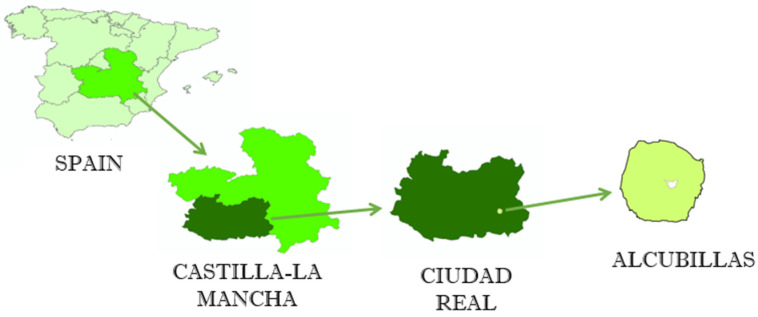
Geographical location of the study area.

The study area corresponds to the municipal term of Alcubillas. This locality (485 inhabitants, 47.46 km^2^ in area) is part of the Spanish Autonomous Community of Castilla-La Mancha (Central Spain), which is shown in Figure 1. The area has the coordinates 38.7579077 N-3.1412646 W. The climate of the area is Mediterranean continental, with maximum rainfall in October, November and April, and minimum in July and August, which are also the warmest months, while the coldest months are December, January and February. The soil moisture regime is xeric [24] with a mesic soil temperature regime, according to soil taxonomy [25].

The main physiographic characteristics of this area include a practically flat or wavy and karstified surface, which is interrupted by residual reliefs in the form of an island (inselberg), and an alluvial plain. The main parent materials of the area can be grouped as follows: (1) metasedimentary Hercynian rocks, which consist of fine-grained quartzose sandstones or quartzites and are free of carbonates, interbedded with shales (usually not cropping); (2) tertiary calcareous rocks composed of limestones (with signs of karstification, as evidenced by the presence of a number of dolinas) and marls; and (3) colluvial Quaternary slope deposits and alluvial deposits (clay-rich sediments) derived from local bedrocks. The studied soils are developed directly on limestones (sometimes karst depressions) or on marls or other sedimentary deposits with the presence of carbonates. A small proportion of the soils are developed on silicic rocks (quartzites or similar) or slope deposits related to these rocks. Finally, other soils are formed by alluvial sediments. These slope deposits and terrace sediments are always weathered.

According to soil taxonomy [25], the local soils are classified as Rhodoxeralf (Typic, Calcic or Petrocalcic), Haploxerepts (Typic) and Calcixerepts (Petrocalcic). Most of the studied soils were red in color (Munsell 2.5YR or redder); therefore, in the area, there is a predominance of red soils (Rhodoxeralf) with calcic or petrocalcic horizons. The land use in the area is mainly agricultural: almost 90% of the area is cultivated with vineyards (Trellis and traditional—goblet vine training—are the usual systems—50% approximately), olive groves, cereals or orchards.

### 2.2. Sampling

Ten soil samples were collected from ten profiles selected from different geomorphological and vineyard types. The samples correspond to Bt or Bw horizons; this type of horizon was selected because most of the water and natural nutritional elements are close to the roots of the grapevines, which develop fundamentally in these types of horizons (approximate depth 25–75 cm). The geographic locations of the sampling sites were recorded with a handheld global GPS device (Table 1). Stones and debris were removed during sampling. The collected samples were stored in the dark and sieved through a 2-mm mesh to separate the coarser fraction. The remaining fine-grained fraction was subsequently homogenized prior to analysis.

### 2.3. Analytical methods

The clay content was determined by the hydrometer method [26]. The total contents of studied trace elements (Ba, Cr, Cu, Pb, Rb, Sr, V and Zr, selected as the most significant or widely employed in viticulture practices) were determined by X-ray fluorescence spectroscopy (XRF) on a Philips PW 2404 spectrophotometer (Philips; Austin, TX, USA) with a maximum power of 4 kW (set of crystal analyzers for LiF220, LiF200, Ge, PET and PX1, flow detector and twinkle detector). This method has been validated for use in geological matrices such as sediments, according to the work of [27]. Quality control was evaluated by duplicate analysis of certified soil reference materials (NIST 2710 and CRM 039). The detection limits are (mg·kg^−1^): Ba 3.86, Cr 0.90, Cu 0.07, Pb 0.06, Rb 0.19, Sr 0.20, V 1.61 and Zr 0.12.

The mineralogical compositions of the bulk samples were determined by powder X-ray diffraction (XRD) on a PANalytical X’Pert PRO X-ray diffractometer (Pananalytical; Davis, CA, USA) fitted with a Cu anode. The operating conditions were 40 mA and 45 kV, with a divergence slit of 0.5° and a reception slit of 0.5 mm. The samples were scanned in (2θ) 0.0167 steps with a counting time of 150 s. The characterization of bulk samples was performed using the random power method, operating from 5° to 60° 2θ [28]. Rutile was used as an internal reference. The patterns obtained in this way were analyzed using the Match v.3 and Rietveld Full Prof software (Hillsboro, OR, USA) with the Inorganic Crystal Structure Database (ICSD) and the Crystallography Open Database (COD) [29]. In order to identify clay minerals, this fraction was extracted and studied by XRD between 3° and 20° under the same conditions as described above. The identification analysis of the minerals in the clay fraction was studied also by X-ray diffraction in the diffractometer used for the total sample study. Preparations of oriented aggregates dried in air, glycollated with ethylene glycol and calcined at 550 °C should be carried out for two hours, and thus illite and kaolinite have been identified.

### 2.4. Data Treatment and Representation

The geographic distribution of elements can be analyzed using the kriging or IDW (inverse distance weight) techniques. The method involving the IDW algorithm was selected because this only takes into account the distance between samples, and it results in a graphical representation closer to the measured data than that obtained by other algorithms, such as geostatistics-based ones. Maps were produced using GIS software ArcGis v.10.2 (ESRI; Redlands, CA, USA) for analysis. Statistical analysis was performed using IBM software SPSS v.24 (Chicago, IL, USA) under an institutional license for the University of Castilla-La Mancha (Spain).

## 3. Results and Discussion

### 3.1. Mineralogical Characteristics

**Figure 2:**
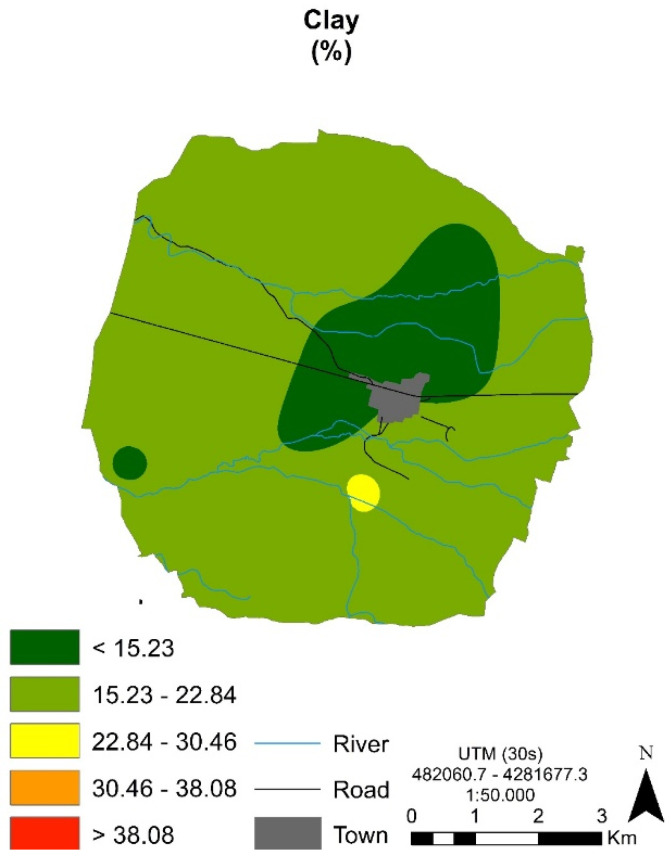
Spatial distribution of textural clay contents.

The percentages of clay contents are shown in Figure 2, along with their geographic distribution. The mineralogical characteristics are provided in Table 2.

**Figure 3:**
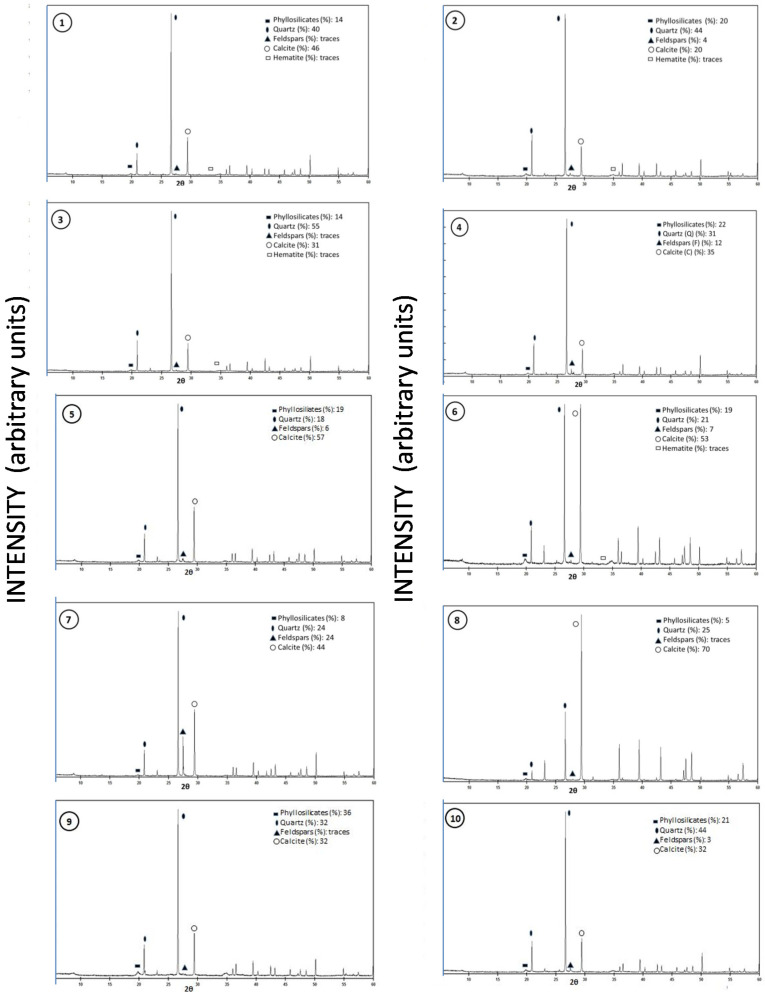
X-ray diffractograms of soil samples; (1, 2, 3, 4, 5, 6, 7, 8, 9, and 10) sample number; black rectangle = phylosilicates; black ellipse = quartz; black triangle = feldspars; circle = calcite; square = hematite.

X-ray diffraction-based characterization of the mineralogy of the area revealed that the studied soils are very homogeneous, with a very similar mineral composition. This mineralogical composition included predominantly quartz and calcite, with a minor generalized presence of phyllosilicates (illite, kaolinite) and feldspars and, in some samples, hematite (Figure 3 and Table 2 and Table 3).

Despite the fact that several geomorphological conditions are present, weathering processes that lead to soil forming act in similar ways over time, to a greater or lesser extent. In all soil profiles studied, the weathering of limestones and marl rocks through the dissolution of CaCO_3_ resulted in the residual accumulation of clay minerals, such as illite (physical breakdown of micas) and kaolinite. As a consequence, the spatial distribution of clay content (Figure 2) and the quantitative relative analysis of clay mineralogical composition displayed a similar variability that depends on some local soil-forming factors and soil management practices (Figure 3). Several authors [4,30,31] reported the predominance of illite and kaolinite in soils of the Mediterranean region. Crystalline quartz, a mineral with high weathering resistance, was found, and this is related with surrounding rocks (quartzites and sandstone quartzites).

The typology and analysis of clay minerals has been widely used to assess weathering intensity [32], since the genetic signals of pedogenic processes leave their mark, amongst others, in the clay mineral composition [33]. In the study reported here, no significant differences were found in the clay mineral composition between different individual soils (Figure 3 and Table 2). In fact, in the examined profiles, the clay composition in Bt (argillic) horizons or Bw (cambic) horizons is marked by a preferential accumulation of illite and kaolinite (Table 3). This is probably a characteristic associated with the climatic conditions of the area, which do not allow the formation of other clay mineral species in the actual conditions of drainage and rock materials.

The local climate, which is characterized by marked differences between dry and rainy seasons, should also favor the release of iron from primary Fe-bearing minerals—after their hydrolysis, oxidation and crystallization of residuals—in the form of hydroxides and oxy-hydroxides (hematite and other related mineral phases), which are responsible for the characteristic red color of these soils [34,35].

The studied soils did not have eluvial horizons, whereas clay illuviation and accumulation of iron oxides (red color) were commonly observed. The process of Fe release and accumulation (accompanied by clay eluviation and illuviation) appears in red Mediterranean soils, but clay illuviation in the strictest sense only occurs at the northern margins of the Mediterranean basin [5].

### 3.2. Geochemistry and Spatial Variability of Studied Trace Elements (Ba, Cr, Cu, Pb, Rb, Sr, V and Zr)

The analyzed elements were found in concentrations within the expected range in Spanish agricultural areas [36], and the concentrations were low when compared with their levels in the Earth’s crust [37]. Most of these contents probably have the same origin, and this is influenced by their contents in geological formations and soil types, although some elements are probably influenced by agricultural activities (or other minor activities in this area). The accumulation of trace elements and, in particular, heavy metals or potentially toxic elements is often the result of the interaction of multiple sources [38].

The statistical parameters for the studied elements are reported in Table 4. The median concentrations for Ba, Cr and Cu were higher than the median values reported for Castilla-La Mancha soils, the values for Sr and Pb were lower than those reported for Castilla-La Mancha soils and, for Rb and Zn, the concentrations were similar to those reported for Castilla-La Mancha soils [16].

**Figure 4:**
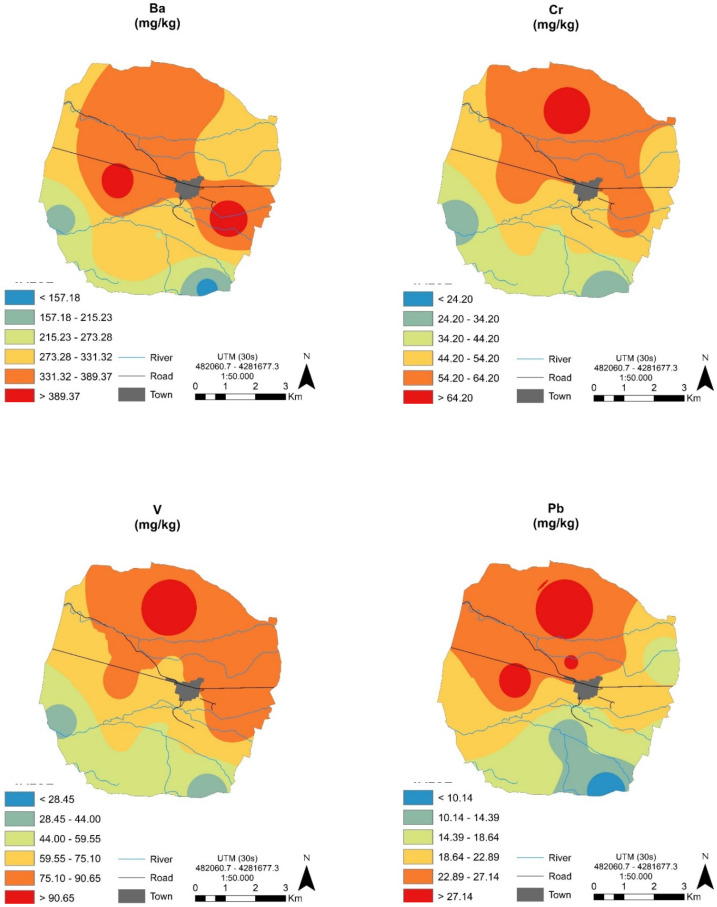

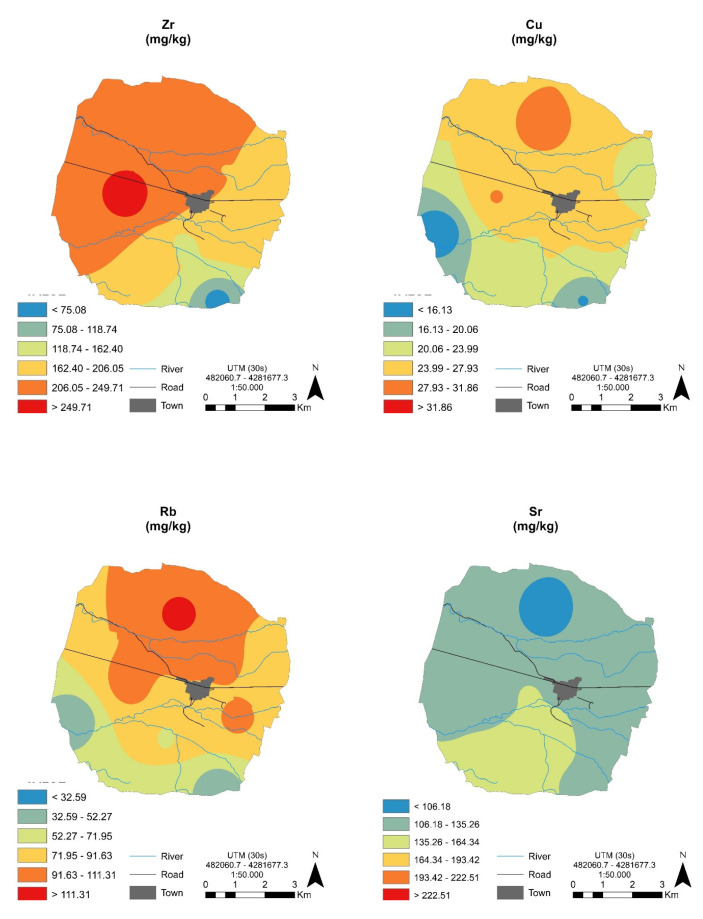
Geo-spatial prediction maps for some trace elements in subsurface soil horizons.

The high degree of spatial variability in the geochemical composition of soils in the world is widely accepted [39]. The mineralogy appears to be relatively uniform in the soils studied here, but we did attempt to clarify the degree of spatial variability of the trace elements analyzed in this zone. The application of geostatistics to soil science has allowed the estimation and mapping of soil attributes in unsampled areas [22,40,41,42]. With this aim in mind, and given that the geostatistical approach has been widely applied to analyze the spatial structure and spatial distribution of trace elements in soil [8,43,44], the geo-spatial distributions (contour maps) were obtained (see Figure 4) for some elements in subsurface soil horizons (B horizons).

The maps obtained (Figure 4) mainly reflect the influence of parent material and soil types on the elemental concentrations in local soils. Nevertheless, the maps also revealed some differences related to this territory in that similar geo-spatial distribution patterns were observed for some elements, i.e., Cr, V, Rb and, to a lesser extent, Ba and Pb. The geo-spatial distribution of Sr indicates a lower geographic variability in comparison with the other elements (Figure 4). Nevertheless, moderate concentrations of some elements were located in certain sites.

The spatial variability of trace elements may be affected by both intrinsic (soil parent materials) and extrinsic (e.g., specific agricultural practices) factors [45]. Linked to background levels, the dependence of the studied trace elements can be attributed to intrinsic properties, and only in particular zones can weak spatial dependencies be attributed to extrinsic aspects. In this respect, the use of chemicals in agriculture is very common, with the main purpose of improving nutrient supply to soils (fertilizers such as urea, calcium superphosphate, iron sulfate and copper sulfate) and disease control (pesticides), as reported by [6,43], amongst others.

Regarding the distribution of Cu concentrations (Table 4), moderate to high values were identified in the north of the studied area (Figure 4), and these were presumably caused by agricultural practices. Based on our research, and considering that there are no industries in the studied area, we can postulate that the relatively high levels of some elements in local sites can be attributed to the use of agrochemicals and irrigation [46,47].

The information presented above on the characterization of elements could be carefully used to diagnose possible deficiencies or imbalances, and also in order to define the fingerprints of vines of this local zone.

Finally, since the contents of analyzed trace elements found in the area do not involve pollution in any case, and because the soils of this zone are principally calcareous in nature (with alkaline pH and low organic matter content), minimal to zero negative impact is expected from these trace elements. In addition, based on the maps obtained and the direct and close relationships with the local farmers, it can be inferred that, despite anthropic pressure over many years, the red vineyard soils of Alcubillas show very little evidence of negative effects. Indeed, it was clearly demonstrated that agricultural production can continue, but some care should be taken to protect the security of agricultural products, including a reduction in the amount of fertilizers and pesticides used in certain areas.

## 4. Conclusions

The study reported here was carried out to provide baseline information on the mineralogy, geochemistry and spatial variability of some trace elements in different soils of Mediterranean typology, in a traditional viticultural area. The mineralogical composition shows minor variations in the studied soils, which are predominantly quartz and calcite, but with illite, kaolinite and feldspars also generally present as minor phases.

In spite of the occurrence of several local geomorphological conditions, soil-forming weathering processes act in similar ways over time, to a greater or lesser extent.

The studied calcareous vineyard soils display a moderately heterogeneous concentration in the analyzed trace elements. The low levels, which are similar to the regional background levels of these elements, and the fact that they show moderately regular spatial distribution patterns, indicate that the elements derive from the soil parent materials and their concentrations mostly depend on soil type. However, local high concentrations are probably related with anthropogenic agricultural practices.

## Figures and Tables

**Table 1:** Some data description of the studied soils; (masl) = meters above sea level.

**Profile**	**1**	**2**	**3**	**4**	**5**
**Location**	0487818 x–4290255 y	0487689 x–4291840 y	0489226 x–4290512 y	0486047 x–4289589 y	0487092 x–4289216 y
**Altitude (masl)**	795	793	801	789	777
**Macromorphology**	Ap-Bt-Ck	Ap-Bt-Ckm	Ap-Bt-Ckg	Ap-Bt-Ckm	Ap-Bw-C
**Soil Type** (Soil Taxonomy 2014)	Calcic Rhodoxeralf	Calcic Rhodoxeralf	Calcic Rhodoxeralf	Petrocalcic Rhodoxeralf	Typic Haploxerept
**Profile**	**6**	**7**	**8**	**9**	**10**
**Location**	0487710 x–4287936 y	0487374 x–4287857 y	0484156 x–4288403 y	0488954 x–4286098 y	0490701 x–4290416 y
**Altitude (masl)**	776	786	812	797	809
**Macromorphology**	Ap-Bw-C	Ap-Bw-C	Ap-Ckm	Ap-Bt-C	Ap-Bt-Ck
**Soil Type** (Soil Taxonomy 2014)	Typic Haploxerept	Typic Haploxerept	Petrocalcic Calcixerept	Calcic Rhodoxeralf	Calcic Rhodoxeralf

**Table 2:** Mineralogical composition (%) of total sample from B horizons by XRD.

Sample	Phyllosilicates (%)	Quartz (%)	Feldspar (%) *	Calcite (%)	Amorphous Materials (%)	R_B_	X^2^
1	14	40	Traces	40	6	17.6	7.3
2	15	44	7	29	5	19.1	7.7
3	14	46	Traces	34	6	22.3	8.2
4	17	31	12*	35	5	23.9	6.9
5	18	19	6	50	7	17.9	6.2
6	22	10	4	54	10	16.8	5.7
7	8	22	20	44	6	19.4	8.1
8	10	25	Traces	50	15	18.6	6.5
9	26	32	Traces	32	10	16.9	6.8
10	21	40	3	32	4	17.5	7.2

* K feldspar and Na, Ca feldspar; R_B_: Bragg R factor; X^2^: Rietveld goodness of fit.

**Table 3:** Mineralogical composition (%) of clay mineral from B horizons by XRD.

Sample	Illite (%)	Kaolinite (%)
1	90	10
2	79	21
3	92	8
4	95	5
5	94	6
6	88	12
7	90	10
8	30	70
9	85	15
10	90	10

**Table 4:** Main statistics for elements (expressed in mg·kg^−1^) analyzed in this study.

(MG/KG)	MEAN	SD	MIN	MAX	MEDIAN CLM SOILS A
**BA**	323.8	69.9	177.4	446.4	214.2
**CR**	51.4	10.9	29.2	71.0	42.7
**CU**	25.0	4.1	17.4	32.4	17.4
**PB**	21.9	6.0	13.3	31.6	25.8
**RB**	83.9	20.9	40.4	118.9	81.2
**SR**	124.4	14.3	101.6	152.8	241.9
**V**	72.5	15.3	45.6	99.0	59.3
**ZN**	47.4	12.3	28.5	72.3	43.5

SD: standard deviation; min: minimum; max: maximum. ^a^ Bravo et al., 2019.
